# Quality and Safety in eHealth: The Need to Build the Evidence Base

**DOI:** 10.2196/16689

**Published:** 2019-12-19

**Authors:** Elizabeth Borycki

**Affiliations:** 1 School of Health Information Science University of Victoria Victoria, BC Canada

**Keywords:** patient safety, technology-induced error, health technology

## Abstract

Research in the area of health technology safety has demonstrated that technology may both improve patient safety and introduce new types of technology-induced errors. Thus, there is a need to publish safety science literature to develop an evidence-based research base, on which we can continually develop new, safe technologies and improve patient safety. The aim of this viewpoint is to argue for the need to advance evidence-based research in health informatics, so that new technologies can be designed, developed, and implemented for their safety prior to their use in health care. This viewpoint offers a historical perspective on the development of health informatics and safety literature in the area of health technology. I argue for the need to conduct safety studies of technologies used by health professionals and consumers to develop an evidence base in this area. Ongoing research is necessary to improve the quality and safety of health technologies. Over the past several decades, we have seen health informatics emerge as a discipline, with growing research in the field examining the design, development, and implementation of different health technologies and new challenges such as those associated with the quality and safety of technology use. Future research will need to focus on how we can continually extend safety science in this area. There is a need to integrate evidence-based research into the design, development, and implementation of health technologies to improve their safety and reduce technology-induced errors.

## Introduction

Health informatics research has made significant contributions to our health care system. From its earliest beginnings in the 1950s and 1960s in Germany and the United States, when health care researchers first began to study the design of software for supporting clinical work and integrating computers into hospitals, to our current work in professionalizing the health informatics and health technology workforce, much work has been done. Today, there is a growing body of research describing a unique area of knowledge (ie, health informatics or biomedical informatics) that has emerged and evolved over the past 70 years [1]. Health informatics (or biomedical informatics) and health technology professionals can be found in every regional health authority, government, and health technology vendor business worldwide. Health informatics (and biomedical informatics) programs are present in many countries worldwide [2]. Nonetheless, many challenges remain as new health technologies are innovated, designed, and implemented and evolve over time. Challenges have arisen from the emergence of quality and safety issues involving newly innovated and existing implemented technologies.

## Health Technologies

### Modernizing Health Care

Technological innovation in health care has modernized how we treat patients globally and how health professionals work across regions and countries [1]. Over the past decades, since the first publications of journal articles and academic texts in the field of health informatics, the body of research and literature has grown considerably [1]. The launch of health informatics journals over the past 20 years has been critical to helping establish our current evidence base and demonstrating the impact of health informatics research internationally on health care and citizens. The design, development, and implementation of new health technologies and innovations remain important areas of study and add to the growth of our discipline and field.

### Considering the Evidence Base

The evolution of knowledge in the area of health informatics has grown considerably. Researchers have designed and developed technologies and studied their implementation. Health technologies such as electronic health records and their subsystems (eg, clinician order entry, laboratory information systems, diagnostic imaging systems, clinical documentation systems, and decision support systems) have improved the quality and safety of health care [1]. Clinician order entry [3] and clinical documentation systems have reduced medical error rates by improving the legibility and readability of clinician orders and activities [4].

Decision support systems have prevented medical errors arising from drug-drug interactions and drug-allergy interactions and have reduced the costs of prescribed medication [1,3]. Laboratory information and diagnostic imaging systems are providing test and imaging results in real time, so that physicians can diagnose diseases and conditions and immediately respond with lifesaving interventions [5,6]. Despite this, we need to continue advancing research in health informatics to study the safety of health informatics in supporting health care processes and its effectiveness in improving wellness, as new technologies are designed and developed or repurposed by health care consumers, health professionals, and health care administrators for use in health care [7,8].

Further research is needed to ensure that technologies provide evidence-based interventions and that health technologies are designed, developed, and tested using accepted, evidence-based research methods, so that they can improve or support the health of individuals, families, and populations [9]. In addition, more research is needed to guide consumers in their purchase of health technologies [7]. To date, research has shown that the purchase of health technologies without any demonstrated health or wellness benefits may lead to unnecessary financial losses or consumer harm in an already vulnerable population [10]. Research has already shown that health technologies, when not properly and safely designed, developed, and implemented, can lead to death and disability, with reports of incidents involving technology (ie, technology-induced errors) increasing worldwide as the number of technologies used to support care increase [11,12]. Several studies focusing on incident reports involving technology-induced errors have identified a link between poorly designed and implemented technologies and safety incidents [13-15]. As such, the need to use health informatics (or biomedical informatics) disciplinary research to support evidence-based technology innovation, design, procurement, implementation, and maintenance by health care administrators, policy makers, and consumers has become critical to ensuring the quality and safety of health care when new technologies are introduced [16,17].

There is a considerable need to continue to conduct research and change health care administrator and policy-maker culture. This involves moving away from the procurement and purchasing of health technologies on the basis of perceived innovations and potential improvements (ie, the latest and greatest technology “fad”) to meaningfully evaluate the quality and safety of a technology before it is implemented and used. There is also a need to move away from studying technologies after they have been designed and developed to examining and studying the quality and safety of a technology during its design and development through to its implementation and use before widespread scale up and spread [16-18]. As research has identified, poor systems design and development processes can lead to consumer death and disability and diminish the effectiveness of the technology intervention where health and wellness are concerned [13,19]. With such knowledge, we can improve the quality of patient care and prevent technology-induced errors that lead to death and disability [13].

## Future Research Directions

In 1992, the seminal publication of “To Err is Human” by the National Academies of Sciences launched health care into the modern world by recommending that health information technologies (eg, clinician order entry) be used to prevent medical error and improve the quality of patient care [20]. By 2005, the first publications emerged for documenting the existence of technology-induced errors [11]. In 2011, the “Health Information Systems Safety” report by the National Academies of Science suggested that we need to develop a strategy for ensuring the quality and safety of health technologies from their design through their development [21]. Today, researchers have found that safety incidents in fully digitized health care systems involve health technologies [13]. Researchers have found that 0.1%-17% of safety incidents involve technology-induced errors [13-15]. Future health technology applications involving artificial intelligence (AI) will also need to be thoroughly innovated and studied to determine if the technology is safe and improves the quality of patient care, health professional work, and health care systems performance. Research identifies that AI does not fully respond to the range of health care issues that a patient may experience and that there is still a need for health professionals to be “in the loop” to validate diagnoses and actions to be carried out by AI applications in different health care contexts [22].

With the introduction of the Internet of Things (IoT), new challenges will emerge in the area of quality and safety. IoT encompasses sensors, devices, wearables, and smartphones. Essentially, these technologies speak to each other via the internet, and it is through these combinations of devices and automated systems that information can be gathered and analyzed and responses can be created to help individuals with tasks or processes [19]. IoT technologies have been found to have some important applications, but also need to be critically studied. There is a need to innovate, design, and develop IoT that is effective and integrates these technologies into the system of patient self-care and the health care system care. New IoT systems are being designed and developed to support those patients with chronic illnesses such as diabetes and those with multiple comorbidities. There is a need to study what devices work best to improve health outcomes while maintaining health safety [23]. Algorithms used in devices and software need to be studied in this context to determine their effectiveness in one health care setting versus another, as reproducibility and reusability have emerged as issues. Such research is needed to study the utility of algorithms and what changes need to be made to the algorithm itself to be effective in a given health context [24]. The integration of robotics in health care also requires extensive study. Innovation involves not only designing and developing the technology, but also identifying and studying the disease contexts, settings, and methods of using robotics to deliver health care interventions (eg, the use of robotic surgery) on patient circumstance and outcomes and to develop safe practices [25].

Finally, there is a need to study the use of existing technologies (ie, mobile health apps, wearable devices that support wellness and fitness) that have been deployed to identify technologies that provide no added benefit or may introduce new risks and to use this evidence in health policy making. Here, the design, development, implementation, and use of these devices needs to be researched [26]. In addition, new ways to educate consumers about these health technologies and ways to best select a health technology that will improve health and wellness rather than mislead consumers to engage in risky behaviors (eg, disregarding physician recommendations, poor medication adherence) are needed. Without research in health informatics (and biomedical informatics), we would not have our present understanding of the evidence base supporting health technologies. More research is needed to inform consumers, health care administrators, and policy makers, so that funds are not spent on ineffective and unsafe technologies. Disseminating this research to administrators and policy makers is essential to begin the process of using decision making for evidence-based health technology, based on the growing health informatics (and biomedical informatics) research.

## Abbreviations

AI: artificial intelligence
IoT: Internet of Things
